# Suicidal Risk Behaviors in Adolescents With Rare Neurodevelopmental Disorders: The Role of Sex, Autistic Traits, and Mental Health Difficulties

**DOI:** 10.1093/jpepsy/jsad051

**Published:** 2023-08-08

**Authors:** Stian Orm, Jeffrey Wood, Blythe Corbett, Krister Fjermestad

**Affiliations:** Division of Mental Health Care, Innlandet Hospital Trust, Norway; Frambu Resource Center for Rare Disorders, Norway; Department of Education, University of California, USA; Department of Psychiatry and Behavioral Sciences, Vanderbilt University Medical Center, USA; Frambu Resource Center for Rare Disorders, Norway; Department of Psychology, University of Oslo, Norway

**Keywords:** anxiety, autism, depression, rare disorders, suicidality

## Abstract

**Objective:**

Autistic traits are associated with mental health difficulties and risk of suicidal risk behaviors among adolescents. Little is known about how autistic traits affect the mental health of adolescents with rare neurodevelopmental disorders (RNDs). The aim of this study was to investigate the relationship between autistic traits, mental health difficulties, and suicidal risk behaviors in adolescents with RNDs.

**Methods:**

Parents (*N *=* *93) completed the Child Behavior Checklist, Social Communication Questionnaire, and Social Responsiveness Scale about their adolescent (*M*_age_ = 13.1, *SD* = 2.3, 62.4% females) with an RND (e.g., sex chromosome aneuploidies, Fragile X syndrome, 22q11.2 deletion syndrome). The data were analyzed with hierarchical logistic regression analysis.

**Results:**

The prevalence of suicidal risk behaviors (16.1%) was similar to that reported among autistic youth and was higher among boys than girls. More autistic traits were associated with suicidal risk behaviors in bivariate analysis. In multivariate analysis, more anxiety/depressive symptoms were associated with more suicidal risk behaviors and externalizing problems associated with suicidal risk behaviors beyond autistic traits and anxiety/depressive symptoms.

**Conclusion:**

Adolescents with RNDs are at risk of suicidal risk behaviors, especially those with higher levels of autistic traits, anxiety/depressive symptoms, and externalizing problems. Assessment of autistic traits, mental health difficulties, and suicide risk may be indicated for adolescents with RNDs to determine if corresponding intervention is needed.

## Introduction

Adolescence is a vulnerable period characterized by an increase in anxiety/depressive symptoms and suicidal risk behaviors (McLaughlin & King, 2015; Nock et al., 2013). Studies have highlighted the importance of identifying risk factors for suicidal risk behaviors among adolescents (Adrian et al., 2016). Anxiety/depressive symptoms are often antecedent to and associated with suicidal risk behaviors in adolescence (Carballo et al., 2020). Furthermore, social difficulties and poor peer relationships have been identified as risk factors for both anxiety/depressive symptoms and suicidal risk behaviors (Adrian et al., 2016; Shore et al., 2018). Children and adolescents with chronic disorders have been reported to have increased risk of anxiety/depressive symptoms and social difficulties (Pinquart & Shen, 2011; Pinquart & Teubert, 2012). Identifying risk factors of suicidal risk behaviors is important for clinical work, as assessment and modification of risk factors prior to the emergence of suicidal risk behaviors can prevent psychological distress and potential deaths.

Recent meta-analytic evidence highlights the alarming rates of suicidal risk behaviors among autistic youth (O’Halloran et al., 2022). Population-based studies have found suicide and suicide attempts to be much more frequent among autistic individuals compared to non-autistic individuals, with a 10-fold risk of dying by suicide among autistic individuals (Cassidy et al., 2022; Hirvikoski et al., 2020; Kõlves et al., 2021). In their meta-analysis, O’Halloran et al. (2022) found that 25% of autistic children, adolescents, and young adults have thoughts, ideas, or ruminations about suicide, and almost 10% have attempted suicide across age groups and informants. Autistic traits (i.e., difficulties with social communication and repetitive and restrictive behavior and thinking) are also associated with suicidal risk behaviors (i.e., suicidal ideation, self-harm, suicide attempts) in the general population of children and adolescents (Chen et al., 2020; Culpin et al., 2018).

Rare neurodevelopmental disorders (RNDs), such as rare copy number variants and sex chromosome aneuploidies, are often associated with autistic traits (Richards et al., 2015). Hence, adolescents with RNDs may also be at higher risk of suicidal risk behaviors. A disorder is *rare* if the population prevalence is less than 1:2,000 (EURODIS—Rare Diseases Europe, 2023). In addition to autistic traits, adolescents with RNDs may have cognitive and somatic issues that can further increase the risk of suicidal risk behaviors (e.g., Biswas & Furniss, 2016; Kidd et al., 2014). Examining and documenting the risk of suicidal risk behaviors in adolescents with RNDs are extremely important in a clinical and public health (i.e., to prevent deaths by suicide) context. However, little is known about the relationship between autistic traits, mental health difficulties, and suicidal risk behaviors among adolescents with RNDs.

The following RNDs were included in the current study: 22q11.2 duplication and deletion syndromes, sex chromosome aneuploidies, 16p11.2 duplication and deletion syndromes, and Fragile X syndrome. We chose these RNDs as they are all associated with social difficulties and a high prevalence (i.e., 10-50%) of autism spectrum disorder (e.g., Green Snyder et al., 2016; Richards et al., 2015; Wenger et al., 2016). Furthermore, all of these RNDs are associated with co-occurring anxiety and depressive symptoms, as well as externalizing problems (e.g., Fjermestad et al., 2022; Hoeffding et al., 2017; Niarchou et al., 2019).

Clinical guidelines have addressed the high prevalence of autistic traits, anxiety/depressive symptoms, and externalizing problems among individuals with RNDs and recommend that these issues are addressed in clinical care (Gravholt et al., 2017; Gross et al., 2015; Groth et al., 2013; Óskarsdóttir et al., 2023). These clinical guidelines recommend broad and regular mental health assessments, and some of the assessment tools recommended include items for assessing suicidal risk behaviors (e.g., Gravholt et al., 2017). However, the low prevalence of RNDs may lead to more barriers to the implementation of clinical guidelines compared to non-rare conditions (Gittus et al., 2023). There are also no general guidelines across RNDs. Reviews have shown that up to half of clinical practitioners report to not follow clinical recommendations (Dobscha et al., 2003; Gittus et al., 2023; Zadro et al., 2019). An earlier review showed that a third of clinicians believed that clinical guidelines were too rigid to fit individual patients (Farquhar et al., 2002). Research also shows that even for specially trained suicide risk assessors, the effects of training diminish substantially over just a few months after training (Reiff et al., 2019). This means that despite the fact that broad mental health assessments, including suicide risk, are mentioned in some guidelines (e.g., Gravholt et al., 2017), clinicians may not know or follow these guidelines, or they may follow them poorly. This means that more knowledge about suicide risk across RNDs is needed, to inform further specification and relevance of guidelines.

To date, surprisingly few studies have investigated suicidal risk behaviors among adolescents with RNDs. In a Danish register study, a nonsignificant increased risk of death by suicide was found among 781 individuals with the sex chromosome aneuploidy Klinefelter syndrome (Bojesen et al., 2004). Another study found a prevalence of 6.4% for suicidal ideation among 172 individuals with 22q11.2 deletion syndrome aged 5–54 years (Green et al., 2009). A recent case report (*N *=* *1) found many suicidal risk behaviors in a patient with the sex chromosome aneuploidy Turner syndrome (Motamedi et al., 2021). Given the scarcity of studies, little is currently known about suicidal risk behaviors in adolescents with RNDs, and more studies are warranted.

Studies of suicide risk in adolescents with RNDs need to consider multiple factors, beyond anxiety/depressive symptoms. First, demographic variables such as age and sex are relevant to consider. In the general population, adolescent girls present with more suicidal risk behaviors (i.e., suicidal ideation, self-harm, and suicide attempts) than boys (who are more likely to die by suicide), and older adolescents more than younger adolescents (Cha et al., 2018). However, in the literature on autistic youth, mixed results have been reported with some studies showing no differences based on age and sex (e.g., La Buissonnière Ariza et al., 2022) while others have found more suicidal risk behaviors in older youths (e.g., O’Halloran et al., 2022). Second, autistic traits and autism-related social difficulties may play a role due to associations with loneliness and anxiety/depressive symptoms (Schwartzman & Corbett, 2020; Wood & Gadow, 2010). Third, beyond anxiety/depressive symptoms, externalizing problems may also increase the risk of suicidal risk behaviors (Soto-Sanz et al., 2019). However, among autistic youth, mixed results have been reported on the relationship between externalizing problems and suicidal risk behaviors (see La Buissonnière Ariza et al., 2022 for a review). Furthermore, the association between externalizing problems and suicidal risk behaviors may depend on sex. In a meta-analysis of typically developing youth, externalizing problems were found to increase the risk of suicidal risk behaviors among boys but not girls (Miranda-Mendizabal et al., 2019). It is important to examine how the different risk factors interact or are independent among adolescents with RNDs, a population where other factors such as somatic illness, lack of treatment and support, and smaller user communities (i.e., because of the rare aspect) may contribute to suicidal risk behaviors.

The current study had several objectives: first to examine the presence of suicidal risk behaviors among adolescents with RNDs; second to examine potential age and sex differences in suicidal risk behaviors among adolescents with RNDs; third to test the hypothesis that more autistic traits and anxiety/depressive symptoms are associated with more suicidal risk behaviors; and finally, we explored if externalizing problems had a contribution in explaining variance in suicidal risk behaviors beyond autistic traits and anxiety/depressive symptoms and whether the association was moderated by sex. We relied on parent reports because parents often are gatekeepers for adolescents’ access to mental health services and previous findings have suggested that parent reports more efficiently identify youth with suicidal risk behaviors compared to youth self-reports (Van Meter et al., 2018). Furthermore, previous studies have questioned the reliability and validity of self-reports of youth with neurodevelopmental disorders because of a tendency to underreport their problems compared with informant reports, perhaps due to limited self-awareness of their own difficulties and lack of awareness about internal states (e.g., Emeh et al., 2018; Wood & Gadow, 2010).

## Methods

### Participants and Procedure

We recruited 93 parents of adolescents with the following RNDs: 22q11.2 duplication/deletion syndrome (*n *=* *37), sex chromosome aneuploidy (*n *=* *30; i.e., 47XXY/Klinefelter syndrome (*n *=* *3), 45X/Turner syndrome (*n *=* *18), 47XYY/Jacobs syndrome (*n *=* *6), Triple X syndrome (*n *=* *3)), 16p11.2 duplication/deletion syndrome (*n *=* *16), or Fragile X syndrome (*n *=* *10). The adolescents were mostly European-White (94.6%, 1.1% Asian, 4.3% mixed) and the majority were females (60.2% vs. 39.8% males). The mean age was 13.2 years (*SD* =* *2.4; range 10–17). The mothers were on average 45.6 years of age (*SD *=* *6.6) and their fathers 47.9 years (SD = 7.1). About half of the parents had higher education (i.e., 1 year or more with college studies; mothers: 54.9%; fathers: 47.4%), 58.1% rated their family economy as good or very good in the last two years, whereas 6.1% rated it as poor or very poor (35.5% as neither bad nor good).

The parents were recruited mainly through a national resource center for rare disorders in Norway, using the patient registry and the center’s social media channels. In addition, the national user associations for the represented RNDs were asked to distribute information about the study to their members. Unfortunately, the broad approach to recruitment prevents us from calculating a response rate, since we do not know exactly how many potential participants were reached through user associations and social media. A link with information about the study, including digital informed consent, was distributed to potential participants. After consenting, the participants were automatically transferred to the online questionnaire comprising items related to demographic variables (reported above), mental health, and autism symptomatology. Thus, all data were collected at the same time. This study was prospectively reviewed and approved by the Regional Committee for Ethics in Health Research and the Norwegian Center for Research Data (i.e., the relevant data protection agency).

### Measures

#### Child Behavior Checklist

The Child Behavior Checklist (CBCL; Achenbach & Rescorla, 2001) was used to measure anxiety/depressive symptoms, externalizing problems, and suicidal risk behaviors. The CBCL comprises 112 items tapping symptoms of mental health problems and is rated on a scale from 0 (absent), 1 (occurs sometimes), to 2 (occurs often). In the current study, we used the subscale anxious/depressed which comprises 13 items tapping symptoms of anxiety and depression and externalizing problems which comprise 35 items tapping rule-breaking and aggressive behavior. Two items tap suicidal risk behaviors: items 18 (self-harm/suicide attempt) and 91 (suicidal thoughts). These two items from the CBCL have been found similarly effective (AUC = 0.85) to detect youth with suicide risk as a clinical interview conducted with both youth and their parents (Van Meter et al., 2018). In fact, the CBCL was significantly more effective in identifying youth with suicide risk compared to both youth self-reports (Area Under the Curve [AUC] = 0.70) and teacher reports (AUC = 0.45). An AUC of 0.85 is generally considered excellent for a diagnostic test (Mandrekar, 2010) and suggests that the two CBCL items are an efficient and valid way of assessing suicidal risk behaviors in youth. In the current study, the two items were summed to a suicidal risk-behavior score. Item 91 (suicidal thoughts) is also included in the anxiety/depressive subscale, so to avoid an inflated correlation between this subscale and our suicidal risk-behavior composite, we removed this item from the anxiety/depressive subscale. The CBCL has demonstrated good psychometric properties (Ivanova et al., 2007). In the current study, the anxious/depressed subscale (α = 0.87), the externalizing problems (α = 0.93), and the suicidal risk-behavior composite (α = 0.66) demonstrated adequate internal consistency.

#### Social Communication Questionnaire

The Social Communication Questionnaire—Lifetime version (SCQ; Rutter et al., 2003) was used to measure lifetime autistic traits since the SCQ includes items on developmental history (e.g., *When he/she was 4 to 5 years of age, did he/she nod at you to say yes?*). The SCQ comprises 40 items rated on a yes (1) or no (0) scale. A cutoff of 15 is recommended when screening for autism spectrum disorder with the SCQ. The SCQ has demonstrated good psychometric properties (Chandler et al., 2007; Rutter et al., 2003). In the current study, the SCQ demonstrated good reliability (KR-20 = 0.86).

#### Social Responsiveness Scale

The Social Responsiveness Scale (SRS; Constantino & Gruber, 2005) was used to measure current autistic traits since the SRS items only focus on the last 6 months (e.g., *Play with peers in an inappropriate manner*). The SRS comprises 65 items rated on a Likert scale from not true (0), sometimes true (1), often true (2), to almost always true (3). A cutoff of 65 for girls and 70 for boys are recommended when screening for autism spectrum disorder with the SRS. The SRS has demonstrated good psychometric properties (Constantino et al., 2003; Constantino & Gruber, 2005). In the current study, the SRS demonstrated excellent reliability (α = 0.96).

### Data Analyses

We used SPSS version 28 for statistical analyses. First, we examined sex differences using the chi-square test of independence and estimated the bivariate correlations between lifetime autistic traits, current autistic traits, anxiety/depressive symptoms, externalizing problems, and suicidal risk behaviors. Since suicidal risk behaviors were positively skewed and had a floor effect (i.e., 83.9% of parents reported no suicidal risk behaviors in their adolescent), we used the nonparametric correlation coefficient, Spearman’s rho (ρ), when estimating the bivariate correlations. Second, we conducted hierarchical logistic regression analyses to examine whether autistic traits, anxiety/depressive symptoms, and externalizing problems were associated with suicidal-risk behaviors. In the first block, we entered the demographic variables, sex and age. In the second block, current autistic traits were added. In the third block, anxiety/depressive symptoms were added. In the fourth block, externalizing problems were added. In the fifth block, an interaction effect between externalizing problems and sex was added to examine whether sex moderated the relationship between externalizing problems and suicidal risk behaviors. We tested for multicollinearity by examining the correlations between the different independent variables in the regression models. All correlation coefficients were ≤0.60, indicating that multicollinearity was not an issue. For the chi-square test of independence and the hierarchical logistic regression analysis, the suicidal risk-behavior variable was dichotomized as those with suicidal risk behaviors (i.e., a score of 1–4 on the 0- to 4-point sum score) versus those without suicidal risk behaviors (i.e., a score of 0). Significant results are reported when *p* ≤ .05.

## Results

### Suicidal Risk Behaviors

Descriptive statistics showed that 28% (*n *=* *26) met the cutoff for autism spectrum disorder on the SCQ and 65% (*n *=* *60) met the cutoff on the SRS. Regarding suicidal risk behaviors, 16.1% (*n *=* *15) of parents reported that their adolescent had suicidal risk behaviors during the last 6 months, whereas 83.9% (*n *=* *78) of parents reported an absence of suicidal risk behaviors. Suicidal thoughts were reported more frequently (16.1%) than self-harm/suicide attempt (4.3%). Parents who reported that their adolescent displayed self-harm/suicide attempt also reported suicidal thoughts. Compared to girls (9%), boys (27%) had a fourfold risk of suicidal risk behaviors (χ^2^ (1, *N *=* *93) = 5.395, *p* = .02, OR = 3.78, 95% CI [1.17, 12.18]). There was no bivariate association between age and suicidal risk behaviors (ρ = 0.02, *p* = .82).

See Table I for an overview of correlations and descriptive statistics for each independent and the dependent variable, and Table II for descriptive statistics separated on the rare disorder groups. There were significant positive correlations between lifetime autistic traits (SCQ), current autistic traits (SRS), anxiety/depressive symptoms, and externalizing problems. Current autistic traits (SRS), anxiety/depressive symptoms, and externalizing problems were significantly and positively correlated with suicidal risk behaviors. Since lifetime autistic traits (SCQ) were not significantly correlated with suicidal risk behaviors, further analyses were conducted with current autistic traits (SRS) only.

**Table I.** Descriptive Statistics and Bivariate Correlations Between Study Variables

Variable	*M*	*SD*	1.	2.	3.	4.
1. SCQ	10.51	6.76				
2. SRS	79.05	31.61	0.74***			
3. CBCL anxious/depressive	7.14	5.37	0.31**	0.51***		
4. CBCL suicidal risk behaviors	0.24	0.63	0.17	0.24*	0.32**	
5. CBCL externalizing problems	9.96	10.13	0.42***	0.60***	0.41***	0.46***

*Note*. CBCL = Child Behavior Checklist; SCQ = Social Communication Questionnaire; SRS = Social Responsiveness Scale.

* *p* < .05.

** *p* < .01.

*** *p* < .001.

**Table II.** Descriptive Statistics for Each Group of Rare Disorders

	22q11.2		Sex chromosome		16p11.2		Fragile X	
Variable	*M*	*SD*	*M*	*SD*	*M*	*SD*	*M*	*SD*
SCQ	10.78	6.33	7.40	6.16	13.13	6.44	14.60	7.26
SRS	83.19	28.08	61.93	32.41	97.13	24.50	86.20	31.45
CBCL anxious/depressive	8.46	6.21	5.60	4.87	7.00	4.86	7.10	3.14
CBCL suicidal risk behaviors	0.16	0.44	0.27	0.58	0.50	1.10	0.00	0.00
CBCL externalizing problems	9.49	9.15	7.10	9.42	18.25	12.16	7.00	4.69

*Note*. 22q11.2 = 22q11.2 copy number variants, sex chromosome = sex chromosome aneuploids; 16p11.2 = 16p11.2 copy number variants; CBCL = Child Behavior Checklist; Fragile X = Fragile X syndrome; SCQ = Social Communication Questionnaire; SRS = Social Responsiveness Scale.

### Correlates of Suicidal Risk Behaviors

Table III shows the results from the hierarchical logistic regression analysis. The first block with sex and age was not significant (χ^2^ (2) = 5.49, *p* = .064, Nagelkerke *R*^2^ = 0.098). Adding current autistic traits in the second block significantly improved the model and explained an additional 6.5% of the variance in suicidal risk behaviors (Δ χ^2^ (3) = 9.37, *p* = .025, Nagelkerke *R*^2^ = 0.163). In this model, being a boy significantly increased the odds of suicidal risk behaviors, while there was no significant effect of current autistic traits. Adding anxiety/depressive symptoms in the third block significantly improved the model and explained an additional 10.2% of the variance in suicidal risk behaviors (Δχ^2^ (2) = 15.72, *p* = .003, Nagelkerke *R*^2^ = 0.265). In this model, being a boy and having more anxiety/depressive symptoms significantly increased the odds of suicidal risk behaviors. Adding externalizing problems in the fourth block significantly improved the model and explained an additional 30.9% of the variance in suicidal risk behaviors (Δ χ^2^ (1) = 38.23, *p* < .001, Nagelkerke *R*^2^ = 0.574). In this model, anxiety/depressive symptoms, externalizing problems, and age significantly increased the odds of suicidal risk behaviors. Adding the interaction between sex and externalizing problems in the fifth block did not significantly improve the model (Δ χ^2^ (1) = 1.55, *p* = .213, Nagelkerke *R*^2^ = 0.593).

**Table III.** Results From Hierarchical Logistic Regression With Autistic Traits, Anxiety/Depressive Symptoms, and Externalizing Problems as Predictors of Suicidal Risk Behaviors

Variable	*B*	SE	Wald	*p*-Value	OR	95% CI
Block 1						
Sex	1.36	0.60	5.08	0.02	3.89	1.19, 12.64
Age	0.06	0.13	0.20	0.66	1.06	0.83, 1.35
Block 2						
Sex	1.21	0.62	3.84	0.05	3.36	1.00, 11.31
Age	0.09	0.13	0.49	0.48	1.10	0.85, 1.42
Autistic traits	0.65	0.34	3.53	0.06	1.91	0.97, 3.73
Block 3						
Sex	1.39	0.66	4.46	0.04	4.00	1.11, 14.49
Age	0.13	0.14	0.84	0.36	1.14	0.87, 1.49
Autistic traits	0.28	0.40	0.49	0.48	1.32	0.61, 2.88
Anxiety/depressive symptoms	0.82	0.34	5.84	0.02	2.27	1.17, 4.41
Block 4						
Sex	0.78	0.86	0.82	0.37	2.17	0.40, 11.72
Age	0.47	0.22	4.34	0.04	1.59	1.03, 2.47
Autistic traits	−1.20	0.74	2.66	0.10	0.30	0.07, 1.28
Anxiety/depressive symptoms	1.23	0.53	5.45	0.02	3.42	1.22, 9.66
Externalizing problems	2.46	0.76	10.39	0.001	11.69	2.62, 52.11
Block 5						
Sex	0.15	1.08	0.02	0.89	1.16	0.14, 9.54
Age	0.50	0.24	4.57	0.03	1.65	1.04, 2.62
Autistic traits	−0.92	0.82	1.26	0.26	0.40	0.08, 1.99
Anxiety/depressive symptoms	1.17	0.56	4.33	0.04	3.21	1.07, 9.61
Externalizing problems	1.84	0.88	4.41	0.04	6.27	1.13, 34.82
Sex × externalizing	1.23	1.05	1.39	0.24	3.43	0.44, 26.66

*Note.* Autistic traits = Social Responsiveness Scale; anxiety/depressive symptoms and externalizing problems = Child Behavior Checklist; CI = Confidence Interval.

## Discussion

The findings of this study enhance our knowledge about suicidal risk behaviors in adolescents with RNDs in several ways. First, the prevalence of parent-reported suicidal risk behaviors in our sample of adolescents with RNDs was similar to the pooled parent-reported prevalence estimate among autistic youths (i.e., 17.3%; O’Halloran et al., 2022). Compared to a sample of not clinically referred youth, using the same CBCL items, 18 and 91(Aitken et al., 2016), our sample reported a much higher prevalence of suicidal ideation (item 91: 16.1% vs. 1.8%) and self-harm/suicide attempt (item 18: 4.3% vs. 0.2%). Compared to a sample of youth clinically referred for gender dysphoria, our sample reported a similar prevalence of suicidal ideation (item 91: 16.1% vs. 19.1%) and self-harm/suicide attempt (item 18: 4.3% vs. 6.6%), suggesting that youth with RNDs are similar to clinically referred populations in the rate of suicidal risk behaviors. Furthermore, compared to a study of autistic youth with comorbid anxiety or obsessive-compulsive disorder, also using CBCL item 91, our study shows a slightly higher prevalence of suicidal ideation (16.1% vs. 12.7%; La Buissonnière Ariza et al., 2022).

The prevalence of suicidal ideation identified in our study is considerably higher than the prevalence reported by Green et al. (2009) in a sample of individuals with 22q11.2 deletion syndrome, and Bojesen et al. (2004) found a nonsignificant increase in suicides among individuals with Klinefelter syndrome. The difference is likely due to differences in methodology and sample. Bojesen et al. (2004) used register data and, thus, only had data on individuals who had actually died from suicide, and not suicidal ideation, self-harm, or suicide attempts. Green et al. (2009) included a broad age range spanning 5–54 years of age, and since suicidal risk behaviors often peak during adolescence and emerging adulthood and is less common in young children and older adults, the broad age range may be the reason for the different results. Future studies should include broad assessments of suicidal risk behaviors, examine the prevalence of each specific behavior, and carefully consider the role of age to resolve the discrepancies in findings.

In contrast to typically developing youth, there was a higher prevalence of suicidal risk behaviors among boys than girls with RNDs. However, this sex difference was not significant in multivariate analyses and requires further investigation. Similarly, we found no bivariate association with age but age was a significant correlate in the final model, suggesting that higher age may increase the odds of suicidal risk behavior among adolescents with RNDs, but this needs further investigation. It may be that the different risk factors are not independent. For example, it could be that the association between sex and suicidal risk behaviors is accounted for by the presence of more externalizing problems in boys versus girls (Demmer et al., 2017). Furthermore, current autistic traits were significantly associated with suicidal risk behaviors in bivariate analyses but not in the final model. Autistic traits are associated with more anxiety/depressive symptoms (Chen et al., 2020; Wood & Gadow, 2010) which may be particularly pertinent in autistic adolescents (Schwartzman et al., 2022; Schwartzman & Corbett, 2020). Adolescents with many autistic traits may be more prone to peer victimization and rejection, which can cause social anxiety and negative affectivity associated with anxiety/depressive symptoms (Wood & Gadow, 2010). Thus, more autistic traits may lead to more anxiety/depressive symptoms which may again predict more suicidal risk behaviors. In fact, a recent population study of children aged 8–14 years showed that anxiety/depressive symptoms statistically mediated the relationship between autistic traits and suicidal risk behaviors (Chen et al., 2020), and another study of autistic youth found no significant effect of autistic traits in a model including anxiety symptoms (La Buissonnière Ariza et al., 2022). However, such a possible developmental pathway must be investigated further in longitudinal studies.

The bivariate association between autistic traits and suicidal risk behaviors suggests, however, that adolescents with RNDs who display more suicidal risk behaviors also experience greater social difficulties. This is in accordance with the interpersonal theory of suicide (Joiner, 2005), which states that thwarted belongingness—a feeling of not belonging and being socially excluded—and perceived burdensomeness—the feeling of being a burden to others—can lead an individual to desire death. It is possible that autism-related social challenges could increase the risk of suicidal risk behaviors by inducing feelings of thwarted belongingness and perceived burdensomeness (e.g., Pelton et al., 2020). However, the findings of our study suggest that anxiety/depressive symptoms and externalizing problems are more proximal and important correlates of suicidal risk behaviors in adolescents with RNDs.

We found that externalizing problems had a contribution to explaining variance in suicidal risk behaviors beyond autistic traits and anxiety/depressive symptoms. There could be several reasons why externalizing problems are such an important contributor to suicidal risk behaviors among adolescents with RNDs. One reason could be that emotional difficulties such as anxiety/depression may have a more externalizing expression in some children with developmental disorders (La Buissonnière Ariza et al., 2022; Wood & Gadow, 2010). These children may be more likely to impulsively express suicidal thoughts. Furthermore, anxiety/depressive symptoms and externalizing problems will often co-occur, and adolescents with co-occurring anxiety/depression and externalizing problems may be at particular risk of suicidal risk behaviors because externalizing problems cause additional conflicts and problems in interpersonal relationships separate from the effect of autism-related challenges (e.g., La Buissonnière Ariza et al., 2022).

Together, our findings suggest that adolescents with RNDs are at high risk of suicidal risk behaviors compared to general population youth and that the risk is similar to that of other clinical populations, such as youth with gender dysphoria and autistic youth. Our findings may have several clinical implications. The foremost is that adolescents with RNDs are at relatively high risk of suicidal risk behaviors. This knowledge is imperative to clinicians and caregivers, as screening for suicidality may be warranted, and if our results are replicated, clinical guidelines for RNDs should highlight the importance of screening specifically for suicidal risk. Our study also suggests that clinicians, in line with current guidelines (e.g., Gravholt et al., 2017; Óskarsdóttir et al., 2023), should assess anxiety/depressive symptoms and externalizing problems in adolescents with RNDs, as these issues are related to suicidal risk behaviors, and more research are needed on how to implement clinical guidelines for RNDs in clinical practice (Gittus et al., 2023). The fact that the effect of autistic traits disappeared in multivariate analyses, whereas anxiety/depressive symptoms and externalizing problems remained significant correlates, suggests that mental health issues could be a primary focus of preventive efforts. That is, anxiety/depressive symptoms and externalizing problems may be more proximal risk factors of suicidal risk behaviors among adolescents with RNDs than autistic traits.

### Strengths and Limitations

A comparatively large and sex-balanced sample of adolescents with RNDs and the use of psychometrically sound and well-established measures are among the strengths of this study. However, several limitations should be considered when interpreting our findings. The sample comprised several different RNDs. Although these disorders share some features such as autism-related social difficulties, there are also differences between them, and the sample size of this study did not allow us to examine potential differences. The broad approach to recruitment prevented us from calculating a response rate, and we do not have information on the number of potential participants declining to participate or the reasons for declining. This makes it difficult to determine how representative the sample is. However, the broad approach to recruitment may have increased the representativeness of the sample compared to a clinical sample that would be the result of recruiting only through the resource centers. The cross-sectional nature of the study is also a caveat against causal interpretations regarding developmental pathways. The fact that all variables were measured simultaneously makes it difficult to conclude about which factors are antecedents and which are consequences. The SRS and SCQ cut-off scores identified a somewhat different group of participants with autistic traits, likely due to their different foci (present vs. lifetime) and response format, although when these scores were used continuously, they were highly correlated in this sample. Because the SCQ and the SRS were used as dimensional variables of autistic traits for hypothesis testing in this study, concerns about the differing subgroups when using cut-off scores are minimal. Finally, our measure of suicidal risk behaviors was a brief measure (as opposed to a more comprehensive clinical assessment) and only included parent reports, which may underestimate the presence of anxiety/depressive symptoms and suicidal risk behaviors in adolescents (Doyle & Fite, 2022). For example, the meta-analysis by O’Halloran et al. (2022) found the pooled prevalence estimate of suicidal risk behaviors from self-reports to be roughly twice the estimate from parent reports (34.0% vs. 17.3%). Furthermore, although the CBCL items previously showed similar diagnostic efficacy as clinical interviews in detecting youth with suicidal risk behaviors (Van Meter et al., 2018), there is a need for studies utilizing more comprehensive clinical assessments of suicidal risk behaviors among youth with RNDs. There is also a need to examine the validity of commonly used screeners of suicidal risk behaviors among youth with RNDs and autistic traits, as current screening tools may not be sufficiently adapted for use with these youths. In conclusion, longitudinal studies utilizing validated self-reports and/or more comprehensive clinical assessment are important to further elucidate the prevalence of suicidal risk behaviors among adolescents with RNDs and possible developmental pathways.

## Conclusion

This study serves as an important start point for future research endeavors into suicidal risk behaviors among adolescents with RNDs. Adolescents with RNDs seem to be at increased risk of suicidal risk behaviors. Autistic traits, anxiety/depressive symptoms, and externalizing problems seem to be risk factors for suicidal risk behaviors in this population. We call for greater attention around suicidal risk behaviors among adolescents with RNDs and stress the importance of assessing autistic traits, mental health difficulties, and suicidality in this population, and the initiation of preventive efforts.

## Data Availability

Data are available upon reasonable request to the corresponding author, limited by the etichal approval and the concent forms participants signed upon participation.
